# The Development of Attention Systems and Working Memory in Infancy

**DOI:** 10.3389/fnsys.2016.00015

**Published:** 2016-03-03

**Authors:** Greg D. Reynolds, Alexandra C. Romano

**Affiliations:** Developmental Cognitive Neuroscience Laboratory, Department of Psychology, University of TennesseeKnoxville, TN, USA

**Keywords:** infancy, visual attention, recognition memory, working memory, event-related potentials, heart rate

## Abstract

In this article, we review research and theory on the development of attention and working memory in infancy using a developmental cognitive neuroscience framework. We begin with a review of studies examining the influence of attention on neural and behavioral correlates of an earlier developing and closely related form of memory (i.e., recognition memory). Findings from studies measuring attention utilizing looking measures, heart rate, and event-related potentials (ERPs) indicate significant developmental change in sustained and selective attention across the infancy period. For example, infants show gains in the magnitude of the attention related response and spend a greater proportion of time engaged in attention with increasing age (Richards and Turner, 2001). Throughout infancy, attention has a significant impact on infant performance on a variety of tasks tapping into recognition memory; however, this approach to examining the influence of infant attention on memory performance has yet to be utilized in research on working memory. In the second half of the article, we review research on working memory in infancy focusing on studies that provide insight into the developmental timing of significant gains in working memory as well as research and theory related to neural systems potentially involved in working memory in early development. We also examine issues related to measuring and distinguishing between working memory and recognition memory in infancy. To conclude, we discuss relations between the development of attention systems and working memory.

## The Development of attention Systems and Working Memory in infancy

What are the mechanisms that support the ability to retain information for a period of time before acting on it? When does this ability emerge in human development? What role does the development of attention play in this process? Answers to these questions are not only important for furthering our understanding of working memory, but are also fundamental to understanding cognitive development at a broader level. We delve into these questions from a developmental cognitive neuroscience perspective with a particular focus on the impact of the development of attention systems on recognition memory and working memory. In the sections that follow, we present a selective review of research in which psychophysiological and neuroscience techniques have been combined with behavioral tasks to provide insight into the effects of infant attention on performance on recognition memory tasks. We begin our review with a focus on infant attention and recognition memory because the combined measures used in this line of work provide unique insight into the influence of sustained attention on memory. To date, this approach has yet to be utilized to examine relations between attention and working memory in early development. In the second half of the article, we review research on working memory in infancy with a focus on studies utilizing behavioral and neuroscience measures (for more exhaustive reviews, see Cowan, 1995; Nelson, 1995; Pelphrey and Reznick, 2003; Rose et al., 2004; Bauer, 2009; Rovee-Collier and Cuevas, 2009). We also focus on recent research findings that shed light on neural systems potentially involved in attention and working memory in infancy (for excellent reviews on attention and working memory relations in childhood, see Astle and Scerif, 2011; Amso and Scerif, 2015). Because the human infant is incapable of producing verbal or complex behavioral responses and also cannot be given instructions on how to perform a given task, by necessity, many of the existing behavioral studies on infant working memory have been built upon look duration or preferential looking tasks traditionally used to tap into infant visual attention and recognition memory. Thus, it is difficult to draw distinct lines when determining the relative contribution of these cognitive processes to performance on these tasks in the infancy period (but see Perone and Spencer, 2013a,b). We conclude with a section examining potential relations between attention and working memory and propose that the development of attention systems plays a key role in the timing of significant gains in working memory observed in the second half of the first postnatal year.

## Infant Visual attention and Recognition Memory

Much of what we know about the early development of visual attention comes from a large body of research on recognition memory in infancy. Because the defining feature of recognition memory is differential responsiveness to novel stimuli in comparison to familiar (or previously viewed) stimuli (Rose et al., 2004), the majority of behavioral research in the area has utilized the visual paired comparison (VPC) task. This task involves the simultaneous presentation of two visual stimuli. Look duration to each stimulus during the paired comparison is measured. Under the framework of Sokolov’s (1963) comparator model, longer looking to a novel stimulus in comparison to a familiar stimulus (i.e., a novelty preference) is indicative of recognition of a fully encoded familiar stimulus. In contrast, familiarity preferences are indicative of incomplete processing and continued encoding of the familiar stimulus. The underlying assumption is that infants will continue to look at a stimulus until it is fully encoded, at which point attention will be shifted toward novel information in the surrounding environment.

Thus, infant look duration has been a widely used and highly informative behavioral measure of infant attention that also provides insight into memory in early development. Findings from these studies indicate that older infants require less familiarization time to demonstrate novelty preferences than younger infants; and within age groups, increasing the amount of familiarization results in a shift from familiarity preferences to novelty preferences (Rose et al., 1982; Hunter and Ames, 1988; Freeseman et al., 1993). Older infants also show evidence of recognition with longer delays between familiarization and testing. For example, Diamond (1990) found that 4-month-olds demonstrate recognition with up to 10 s delays between familiarization and testing, 6-month-olds demonstrate recognition with up to 1 min delays, and 9-month-olds demonstrate recognition with up to 10 min delays. These findings indicate that with increasing age, infants are able to process visual stimuli more efficiently and subsequently recognize those stimuli after longer delays. Unfortunately for infancy researchers, look duration and attention are not isomorphic. For example, it is not uncommon for infants to continue looking at a stimulus when they are no longer actively paying attention; therefore, looking measures alone do not provide a particularly accurate measure of infant attention. This phenomenon is most prevalent in early infancy and has been referred to as attention capture, obligatory attention, and sticky-fixation (Hood, 1995; Ruff and Rothbart, 1996).

Richards and colleagues (Richards, 1985, 1997; Richards and Casey, 1992; Courage et al., 2006; for review, Reynolds and Richards, 2008) have utilized the electrocardiogram to identify changes in heart rate that coincide with different phases of infant attention. During the course of a single look, infants will cycle through four phases of attention—stimulus orienting, sustained attention, pre-attention termination, and attention termination. The most relevant of these phases are sustained attention and attention termination. Sustained attention is manifested as a significant and sustained decrease in heart rate from prestimulus levels that occurs when infants are actively engaged in an attentive state. Attention termination follows sustained attention and is manifested as a return of heart rate to prestimulus levels. Although the infant is still looking at the stimulus during attention termination, she/he is no longer engaged in an attentive state. Infants require significantly less time to process a visual stimulus if heart rate is measured online and initial exposure is given during sustained attention (Richards, 1997; Frick and Richards, 2001). In stark contrast, infants given initial exposure to a stimulus during attention termination do not demonstrate evidence of recognition of the stimulus in subsequent testing (Richards, 1997).

## The General Arousal/Attention System

Richards (2008, 2010) has proposed that sustained attention is a component of a general arousal system involved in attention. Areas of the brain involved in this general arousal/attention system include, the reticular activating system and other brainstem areas, thalamus, and cardio-inhibitory centers in frontal cortex (Reynolds et al., 2013). Cholinergic inputs to cortical areas originating in the basal forebrain are also involved in this system (Sarter et al., 2001). Activation of this system triggers cascading effects on the overall state of the organism which foster an optimal range of arousal for attention and learning. These effects include: decreased heart rate (i.e., sustained attention), motor quieting, and release of acetylcholine (ACh) via corticopetal projections. Ruff and Rothbart (1996) and Ruff and Capozzoli (2003) description of “focused attention” in children engaged in toy play as being characterized by motor quieting, decreased distractibility, and intense concentration coupled with manipulation/exploration would be considered a behavioral manifestation of this general arousal/attention system.

The general arousal/attention system is functional in early infancy but shows considerable development across infancy and early childhood with increased magnitude of the HR response, increased periods of sustained attention, and decreased distractibility occurring with increasing age (Richards and Cronise, 2000; Richards and Turner, 2001; Reynolds and Richards, 2008). These developmental changes most likely have a direct influence on performance on working memory tasks. The general arousal/attention system is non-specific in that it functions to modulate arousal regardless of the specific task or function the organism is engaged in. The effects of the system on arousal and attention are also general and do not vary in a qualitative manner depending on cognitive task, thus sustained attention would be expected to influence recognition memory and working memory in a similar manner. This non-specific attention system directly influences functioning of three specific visual attention systems that also show considerable development in the infancy period. These specific attention systems are: the reflexive system, the posterior orienting system, and the anterior attention system (Schiller, 1985; Posner and Peterson, 1990; Johnson et al., 1991; Colombo, 2001).

## The Development of attention Systems in The Brain

At birth, newborn visual fixation is believed to be primarily involuntary, exogenously driven, and exclusively under the control of a reflexive system (Schiller, 1985). This reflexive system includes the superior colliculus, the lateral geniculate nucleus of the thalamus, and the primary visual cortex. Many newborn fixations are reflexively driven by direct pathways from the retina to the superior colliculus (Johnson et al., 1991). Infant looking is attracted by basic but salient stimulus features processed via the magnocellular pathway that can generally be discriminated in the peripheral visual field, such as high-contrast borders, motion, and size.

**Figure 1:**
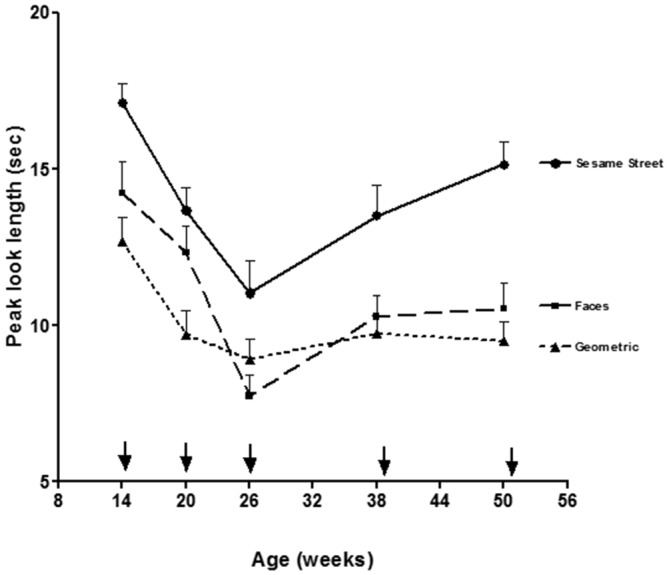
**Mean peak look durations for faces, geometric patterns, and Sesame Street as a function of age (figure adapted from Courage et al., 2006).** Arrows indicate exact test age.

Looking and visual fixation stays primarily reflexive for the first 2 months until the end of the newborn period when the posterior orienting system reaches functional onset. The posterior orienting system is involved in the voluntary control of eye movements, and shows considerable development from 3 to 6 months of age. Areas of the brain involved in the posterior orienting system include: posterior parietal areas, pulvinar, and frontal eye-fields (Posner and Peterson, 1990; Johnson et al., 1991). The posterior parietal areas are believed to be involved in disengaging fixation and the frontal eye-fields are key for initiating voluntary saccades. In support of the view that the ability to voluntary disengage and shift fixation shows significant development across this age range, Figure 1 shows results from a look duration study by Courage et al. (2006) in which infant look duration dropped significantly to a wide range of stimuli from 3 to 6 months of age (i.e., 14–26 weeks of age).

At around 6 months of age, the anterior attention system reaches functional onset and infants begin the drawn out process of developing inhibitory control and higher order attentional control (i.e., executive attention). Not only do infants have better voluntary control over their visual fixations, they can now inhibit attention to distractors and maintain attention for more prolonged periods when it is called for. As can be seen in Figure 1, Courage et al. (2006) found that from 6 to 12 months of age (i.e., 20–52 weeks), infants continue to show brief looks to basic, geometric patterns but begin to show longer looking toward more complex and engaging stimuli such as Sesame Street or human faces. This indicates the emergence of some rudimentary level of attentional control at around 6 months of age. Given that several models emphasize some aspect of attentional control as a core component of working memory (e.g., Baddeley, 1996; Kane and Engle, 2002; Klingberg et al., 2002; Cowan and Morey, 2006; Astle and Scerif, 2011; Amso and Scerif, 2015), it stands to reason that the emergence of attentional control at around 6 months of age would contribute significantly to the development of working memory.

The theoretical models for the attention systems discussed above are largely based on findings from comparative research with monkeys, adult neuroimaging studies, or symptomology of clinical patients with lesions to certain areas of the brain. Unfortunately, developmental cognitive neuroscientists are highly limited in non-invasive neuroimaging tools available for use in basic science with infant participants. However, we have conducted multiple studies utilizing event-related potentials (ERPs) along with heart rate measures of attention and behavioral measures of recognition memory (Reynolds and Richards, 2005; Reynolds et al., 2010). Findings from these studies provide insight into potential areas of the brain involved in attention and recognition memory in infancy.

**Figure 2:**
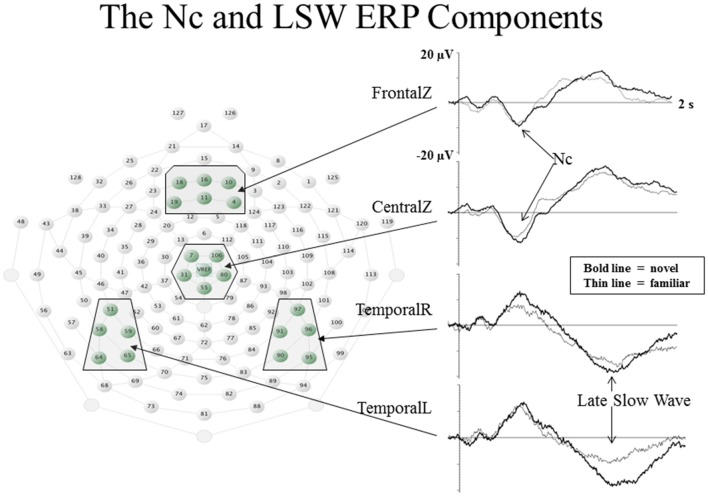
**Event-related potential (ERP) waveforms and electrode locations for the Nc and late slow wave (LSW) ERP components.** The ERP waveforms are shown to the right. Change in amplitude of the ERP from baseline values is represented on the *Y*-axis, and time following stimulus onset is represented on the *X*-axis. The electrode locations for each of the waveforms are shown to the left in boxes on the layout of the EGI 128-channel sensor net (figure adapted from Reynolds et al., 2011).

The ERP component which is most clearly related to infant visual attention is the Negative central (Nc) component. The Nc is a high amplitude, negatively-polarized component that occurs from 400 to 800 ms post stimulus onset at frontal and midline leads (see Figure 2). Nc has been found to be greater in amplitude to: oddball compared to standard stimuli (Courchesne et al., 1981), novel compared to familiar stimuli (Reynolds and Richards, 2005), mother’s face compared to a stranger’s face (de Haan and Nelson, 1997), and a favorite toy compared to a novel toy (de Haan and Nelson, 1999). These findings indicate that regardless of novelty or familiarity, Nc is greater in amplitude to the stimulus that grabs the infant’s attention the most (Reynolds et al., 2010). Additionally, Nc is greater in amplitude when infants are engaged in sustained attention (as measured by heart rate) than when infants have reached attention termination (Richards, 2003; Reynolds et al., 2010; Guy et al., in press). The Nc is also ubiquitous in ERP research utilizing visual stimuli with infant participants. Taken together, these findings indicate that Nc reflects amount of attentional engagement.

**Figure 3:**
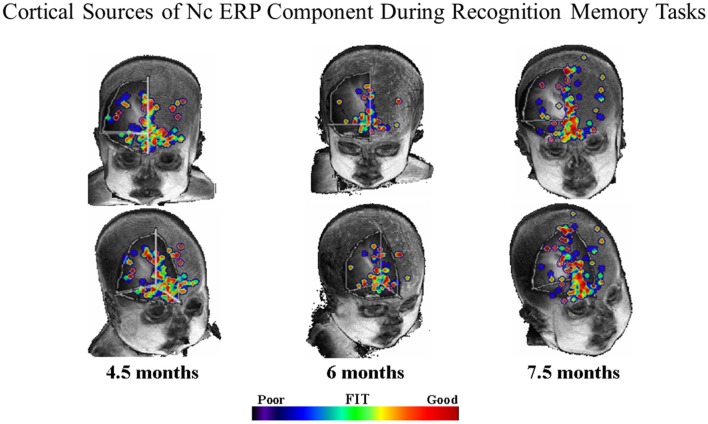
**Common equivalent current dipoles activated across recognition memory tasks.** Age groups are divided into separate columns. The best fitting areas in common between the ERP and visual paired comparison (VPC) tasks are indicated using the color scale. The majority of best fitting areas were located in inferior prefrontal regions (figure adapted from Reynolds et al., 2010).

In order to determine the cortical sources of the Nc component. Reynolds and Richards (2005) and Reynolds et al. (2010) conducted cortical source analysis on scalp-recorded ERP. Cortical source analysis involves computing a forward solution for a set of dipoles, and comparing the simulated topographical plots produced by the forward solution to the topographical plots obtained from observed data. The forward solution is iterated until the best fitting solution is found. The results of the cortical source analysis can then be mapped onto structural MRIs. Figure 3 shows the results of our source analysis of the Nc component measured during brief stimulus ERP presentations and also during performance of the VPC task. As can be seen in Figure 3, the cortical sources of the Nc were localized to areas of prefrontal cortex (PFC) for all age groups including 4.5-month-olds. Areas which were common dipole sources included inferior and superior PFC, and the anterior cingulate. The distribution of the dipoles also became more localized with increasing age. These findings support the proposal that PFC is associated with infant attention, and indicate that there is overlap in brain areas involved in both recognition memory and working memory tasks. Neuroimaging research with older children and adults indicates that there is a neural circuit including parietal areas and PFC involved in working memory (e.g., Goldman-Rakic, 1995; Fuster, 1997; Kane and Engle, 2002; Klingberg et al., 2002; Crone et al., 2006).

The late slow wave (LSW) ERP component is associated with recognition memory in infancy. The LSW shows a reduction in amplitude with repeated presentations of a single stimulus (de Haan and Nelson, 1997, 1999; Reynolds and Richards, 2005; Snyder, 2010; Reynolds et al., 2011). As shown in the two lower ERP waveforms in Figure 2, the LSW occurs from about 1–2 s post stimulus onset at frontal, temporal, and parietal electrodes. By examining the LSW, Guy et al. (2013) found that individual differences in infant visual attention are associated with utilization of different processing strategies when encoding a new stimulus. Infants who tend to demonstrate brief but broadly distributed fixations (referred to as short lookers; e.g., Colombo and Mitchell, 1990) during exposure to a novel stimulus subsequently showed evidence of discriminating hierarchical patterns based on changes in the overall configuration of individual elements (or local features). In contrast, infants who tend to demonstrate longer and more narrowly distributed visual fixations (referred to as long lookers) showed evidence of discriminating patterns based on changes in local features but not based on changes in the overall configuration of local features. Furthermore, research utilizing heart rate measures of attention during performance on a recognition memory ERP task have provided informative findings regarding relations between attention and memory. Infants are more likely to demonstrate differential responding to familiar and novel stimuli in the LSW when heart rate indicates they are engaged in sustained attention (Richards, 2003; Reynolds and Richards, 2005).

No studies to date have utilized cortical source analysis to examine cortical sources of the LSW. Late-latency and long duration ERP components can be more problematic for cortical source analysis due to greater variability in the timing of the latency of the component across participants and trials, and the likely contribution of multiple cortical sources to the ERP component observed in the scalp-recorded EEG. However, research with non-human primates and neuroimaging studies with older children and adults indicates the role of a medial temporal lobe circuit in recognition memory processes. Cortical areas involved in this circuit include the hippocampus and parahippocampal cortex; entorhinal and perirhinal cortices; and the visual area TE (Bachevalier et al., 1993; Begleiter et al., 1993; Fahy et al., 1993; Li et al., 1993; Zhu et al., 1995; Desimone, 1996; Wiggs and Martin, 1998; Xiang and Brown, 1998; Wan et al., 1999; Brown and Aggleton, 2001; Eichenbaum et al., 2007; Zeamer et al., 2010; Reynolds, 2015). Regardless of the potential areas involved in recognition memory in infancy, attention is clearly an integral component of successful performance on recognition memory tasks. Performance on recognition memory tasks is influenced by the development of each of the attention systems described above and it stands to reason that these attention systems would influence performance on working memory tasks in a similar manner. Furthermore, working memory and recognition memory are closely related and some of the tasks used to measure maintenance of items in working memory (i.e., visual short term memory, VSTM) in infancy are slightly modified recognition memory tasks. Thus, distinctions between working memory and recognition memory can be particularly difficult to make during the infancy period.

## The Development of Working Memory in Infancy

Similar to work on attention and recognition memory, research on the early development of working memory has focused on the use of behavioral measures (looking and reaching tasks) with infant participants. Neuroscience models of early working memory development have also largely relied on findings from comparative research, clinical cases, and neuroimaging with older children and adults. However, there is a rich and growing tradition of cognitive neuroscience models and research on working memory development. In the sections that follow, we focus specifically on developmental cognitive neuroscience research on working memory in infancy (for more exhaustive reviews on memory development, see Cowan, 1995; Nelson, 1995; Pelphrey and Reznick, 2003; Courage and Howe, 2004; Rose et al., 2004; Bauer, 2009; Rovee-Collier and Cuevas, 2009).

Much of the research on working memory in infancy has focused on tasks similar to the Piagetian A-not-B task, and generally all tasks involve some delayed response (DR) with the correct response requiring some level of attentional control. The A-not-B and other DR tasks typically involve the presentation of two or more wells. While the participant watches, an attractive object is placed in one of the wells and the participant’s view of the object is then occluded. Following a brief delay, the participant is allowed to retrieve the object from one of the wells. In the A-not-B task, after multiple successful retrieval trials, the location of the hidden object is reversed (again while the participant observes). The classic A-not-B error occurs when the participant continues to reach for the object in the original hiding location after observing the reversal of the hiding location.

Diamond (1985, 1990) has attributed perseverative reaching on the A-not-B task to a lack of inhibitory control in younger participants and attributes higher success rates in older infants (8–9 months) to further maturation of dorsolateral prefrontal cortex (DLPFC). It has been noted (Diamond, 1990; Hofstadter and Reznick, 1996; Stedron et al., 2005) that participants occasionally look to the correct location after reversal but continue to reach to the incorrect (previously rewarded) location. Hofstadter and Reznick (1996) found that when gaze and reach differ in direction, infants are more likely to direct their gaze to the correct location. Thus, poor performance in the A-not-B reaching task may be influenced by immature inhibitory control of reaching behavior as opposed to a working memory deficiency. Alternatively, Smith et al. (1999) conducted a systematic series of experiments using the A-not-B task and found that several factors other than inhibition contribute to perseverative reaching; including infant posture, direction of gaze, preceding activity, and long-term experiences in similar tasks. However, using an oculomotor version of the DR task, Gilmore and Johnson (1995) found that infants as young as 6 months of age were able to demonstrate successful performance. Similarly, using a peek-a-boo looking version of the DR task, Reznick et al. (2004) found evidence of a developmental transition at around 6 months of age associated with improved working memory performance.

In several studies utilizing looking versions of the DR task, significant development has been found to occur from 5 to 12 months of age. With increasing age, infants show higher rates of correct responses, and infants can tolerate longer delays and still demonstrate successful responses (Hofstadter and Reznick, 1996; Pelphrey et al., 2004; Cuevas and Bell, 2010). Bell and colleagues (e.g., Bell and Adams, 1999; Bell, 2001, 2002, 2012; Bell and Wolfe, 2007; Cuevas and Bell, 2011) have integrated EEG measures in looking versions of the A-not-B task in a systematic line of work on the development of working memory. Bell and Fox (1994) found developmental change in baseline frontal EEG power was associated with performance improvement on the A-not-B task. Power changes from baseline to task in the 6–9 Hz EEG frequency band also correlate with successful performance for 8-month-old infants (Bell, 2002). Additionally, higher levels of frontal-parietal and frontal-occipital EEG coherence as well as decreased heart rate from baseline to task are all associated with better performance on the looking version of the A-not-B task (Bell, 2012).

Taken together, these findings provide support for the role of a frontal-parietal network in working memory tasks in infancy which is consistent with findings from neuroimaging studies with older children and adults showing recruitment of DLPFC, ventrolateral prefrontal cortex (VLPFC), intraparietal cortex, and posterior parietal cortex (Sweeney et al., 1996; Fuster, 1997; Courtney et al., 1997; D’Esposito et al., 1999; Klingberg et al., 2002; Crone et al., 2006; Scherf et al., 2006). For example, Crone et al. (2006) utilized fMRI during an object working memory task with children and adults and found that VLPFC was involved in maintenance processes for children and adults, and DLPFC was involved in manipulation of items in working memory for adults and children older than 12. The youngest group of children tested (8–12 years of age) did not recruit DLPFC during item manipulation, and did not perform as well as adolescents and adults on the task.

The change-detection task is used to examine capacity limits for number of items an individual can maintain in VSTM, and the analogous change-preference task is used to measure capacity limits with infant participants. Similar to the VPC task, the change-preference task capitalizes on infants’ tendency to prefer novel or familiar stimuli. Two sets of stimuli are briefly and repeatedly presented to the left and right of midline with items in one set of stimuli changing across each presentation and items in the other set remaining constant. Infant looking to the left and right stimulus set is measured and greater looking to the changing set side is utilized as an index of working memory. Set size is manipulated to determine capacity limits for participants of different ages. Ross-Sheehy et al. (2003) found a capacity increase from 1 to 3 items across 6.5–12.5 months of age. The authors proposed that the increase in capacity limits on this task across this age range is driven in part by development of the ability to bind color to location. In a subsequent study, the authors (Ross-Sheehy et al., 2011) found that providing infants with an attentional cue facilitated memory for items in a stimulus set. Ten month-olds demonstrated enhanced performance when provided with a spatial cue and 5-month-olds demonstrated enhanced performance when provided with a motion cue. These findings demonstrate that spatial orienting and selective attention influences infant performance on a VSTM task, and support the possibility that further development of the posterior orienting system influences maintenance processes involved in working memory in infancy.

Spencer and colleagues (e.g., Spencer et al., 2007; Simmering and Spencer, 2008; Simmering et al., 2008; Perone et al., 2011; Simmering, 2012) have utilized dynamic neural field (DNF) models to explain developmental changes in the change-preference task. Using the DNF model, Perone et al. (2011) did simulation tests of the spatial precision hypothesis (SPH), predicting that the increased working memory capacity limits found to develop during infancy are based on the strengthening of excitatory and inhibitory projections between a working memory field, perceptual field, and an inhibitory layer. According to the DNF model, the perceptual field consists of a population of neurons with receptive fields for certain feature dimensions (e.g., color, shape), and activation in the working memory layer leads to inhibition of similarly tuned neurons in the perceptual field. The results of their simulation experiments were very similar to past behavioral findings and provided support for the SPH in explaining the increases in capacity limits that have been found to occur with increasing age in infancy.

Findings from studies utilizing the change-preference task provide insight into capacity limits in VSTM during infancy. However, this task simply requires identification of novel items or objects based on maintenance of a memory representation over very brief delays (i.e., less than 500 ms). Given that delays between familiarization and testing on infant recognition memory tasks are typically very brief and the length of the delay is often not specified, it is particularly difficult to determine whether or not recognition memory performance is based on short-term memory or long-term memory. Recall that 4-month-olds only demonstrate recognition with up to 10 s delays (Diamond, 1990). Thus, it is also difficult to determine whether or not performance on the change-preference task taps into maintenance of items in working memory or simply measures recognition memory. Alternatively, one could argue that performance on recognition memory tasks with brief delays may be driven by working memory. Interestingly, Perone and Spencer (2013a,b) again utilized the DNF model to simulate infant performance on recognition memory tasks. The results of the simulations indicated that increasing the efficiency of excitatory and inhibitory interactions between the perceptual field and a working memory field in their model led to novelty preferences on VPC trials with less exposure to the familiar stimulus. These simulated results are similar to the developmental trends found to occur with increasing age across infancy in empirical studies utilizing the VPC task (e.g., Rose et al., 1982; Hunter and Ames, 1988; Freeseman et al., 1993). The authors concluded that development of working memory is a significant factor in the increased likelihood that older infants will demonstrate novelty preferences on recognition memory tasks when compared to younger infants.

In order to investigate working memory in infancy, Káldy and Leslie (2003, 2005) conducted a series of experiments with infants that involved both identification and individuation for successful performance. Individuation involves item or object identification combined with entering the identified information into existing memory representations. Infants were familiarized with two objects of different shapes presented repeatedly in the middle of a stage. The side position of the objects was alternated across presentations in order to require infants to integrate object shape with location on a trial by trial basis. During the test phase, the objects were presented in the center of the stage as in familiarization and then placed behind occluders on the same side of the stage. After a delay, the occluders were removed. On change trials, removal of the occluders revealed that the different shaped objects were reversed in location. On no-change control trials, the objects remained in the same location upon removal of the occluders. Longer looking on change trials indicated individuation of the object based on identifying the change in object shape from the location it was in prior to occlusion. Results indicated that while 9-month-olds could identify changes in object location for both objects (Káldy and Leslie, 2003), 6-month-olds were only able to bind object to location for the last object that was moved behind the occluder in the test phase (Káldy and Leslie, 2005). The authors concluded that the younger infants’ memory maintenance was more susceptible to distraction of attention. Káldy and Leslie (2005) also proposed that the significant improvements on this task between 6–9 months of age are related to further development of medial temporal lobe structures (i.e., enthorhinal cortex, parahippocampal cortex) which allows older infants to continue to hold objects in working memory in the presence of distractors.

Thus, Káldy and Leslie (2003, 2005) and Káldy and Sigala (2004) have proposed an alternative model of working memory development which emphasizes the importance of medial temporal lobe structures more so than PFC. They argue that the majority of working memory models emphasizing the importance of DLPFC for working memory are confounding the response inhibition required in typical working memory tasks (e.g., the A-not-B task) with true working memory processes. To further address this limitation, Kaldy and colleagues (Káldy et al., 2015) designed a delayed match retrieval task which involves location-object binding but requires less response inhibition than the classic version of the A-not-B task. Infants are shown two cards, each with pictures of different objects or patterns on them. The cards are turned over and then a third card is placed face up which matches one of the face down cards. Infants are rewarded with an attractive stimulus for looks toward the location of the matching face down card. The authors tested 8- and 10-month-olds on this task and found the 10-month-olds performed significantly above chance levels. Eight month-olds performed at chance levels but showed improvement across trials. Thus, similar to previous work, significant gains in working memory performance are found to occur in the second half of the first postnatal year on the delayed match retrieval task.

Regarding Káldy and Sigala (2004) view that too much emphasis has been placed on the importance of PFC for infant working memory, results from the DNF simulations done by Perone et al. (2011) also support the possibility that areas involved in visual processing and object recognition could account for successful working memory performance on the change-preference task without requiring significant PFC contributions to attentional-control. However, in recent exploratory studies utilizing functional near infrared spectroscopy (fNIRS) to measure the BOLD response of infant participants during an object-permanence task. Baird et al. (2002) observed activation of frontal areas for infant participants during the task. However, receptors were only applied to frontal sites, thus limiting the conclusion that the increased frontal activity during this task was unique or of particular functional significance in comparison to other brain regions. However, Buss et al. (2014) utilized fNIRS to image cortical activity associated with visual working memory capacity in 3- and 4-year-old children. In this study, receptors were applied over frontal and parietal locations. Frontal and parietal channels in the left hemisphere showed increased activation when working memory load was increased from 1 to 3 items. Results supported the possibility that young children utilize a frontal-parietal working memory circuit similar to adults. Both of these findings from fNIRS studies provide preliminary support for the role of PFC in working memory during early development.

Luciana and Nelson (1998) emphasize the critical role the PFC plays in integrating sensorimotor traces in working memory to guide future behavior. According to Luciana and Nelson, the A-not-B task may actually overestimate the functional maturity of the PFC in infant participants because it does not require the accurate integration of sensorimotor traces in working memory. They propose the integration of sensorimotor traces should be considered a core process in working memory definitions. The majority of working memory definitions include executive control components, and persistent activity in DLPFC has been linked with control functions involved in the manipulation of information for the purpose of goal-directed action (e.g., Curtis and D’Esposito, 2003; Crone et al., 2006). Thus, the exact contribution of PFC to working memory functions in early development remains unclear. What is clear from the extant literature is that infants beyond 5–6 months of age are capable of demonstrating basic yet immature aspects of working memory, and significant improvement in these basic functions occurs from 5–6 months (e.g., Diamond, 1990; Gilmore and Johnson, 1995; Hofstadter and Reznick, 1996; Káldy and Leslie, 2003, 2005; Káldy and Sigala, 2004; Pelphrey et al., 2004; Reznick et al., 2004; Cuevas and Bell, 2010).

## The Development of Attention Systems and Working Memory

Similar to recognition memory, the improvements in working memory performance which occur after 5–6 months of age are likely influenced by further development of the attention systems previously discussed. The majority of the working memory studies discussed above examined visuospatial working memory. Performance on all of these working memory tasks involves voluntary eye movements and controlled scanning of the stimuli involved in the task. Thus, functional maturity of the posterior orienting system would be key for successful performance on these tasks. This system shows significant development from 3 to 6 months of age (Johnson et al., 1991; Colombo, 2001; Courage et al., 2006; Reynolds et al., 2013). This timing coincides with the time frame at which infants begin to demonstrate above chance performance on working memory tasks. For example, Gilmore and Johnson (1995) reported successful performance on an oculomotor DR task for 6-month-old infants, and Reznick et al. (2004) describe 6 months of age as a time of transition for performance on a peek-a-boo version of the DR task.

Successful performance on working memory tasks involves more than just voluntary control of eye movements. Working memory tasks also involve attentional control and inhibition. These cognitive functions are both associated with the anterior attention system (Posner and Peterson, 1990), which shows significant and protracted development from 6 months on. Several studies have shown significant improvement on DR and change-preference tasks from 5 to 12 months of age (Hofstadter and Reznick, 1996; Ross-Sheehy et al., 2003; Pelphrey et al., 2004; Cuevas and Bell, 2010), an age range that overlaps with the functional onset of the anterior attention system. Given that some models emphasize the role of PFC and attentional control as being critical for working memory (e.g., Baddeley, 1996; Kane and Engle, 2002; Klingberg et al., 2002), further development of the anterior attention system would be critical for working memory development (for further discussion of attention and memory relations in childhood and adulthood, see Awh and Jonides, 2001; Awh et al., 2006; Astle and Scerif, 2011; Amso and Scerif, 2015).

The general arousal/attention system shows significant developmental change across infancy and early childhood characterized by gains in both the magnitude and duration of periods of sustained attention (Richards and Cronise, 2000; Richards and Turner, 2001; Reynolds and Richards, 2008). Infants are more likely to demonstrate evidence of recognition memory if initial exposure to the test stimulus occurs during sustained attention or if the infant is engaged in sustained attention during the recognition test (e.g., Richards, 1997; Frick and Richards, 2001; Reynolds and Richards, 2005; Reynolds et al., 2010). It stands to reason that these developmental gains in sustained attention would also facilitate improved performance on working memory tasks. This reasoning is supported by Bell (2012) finding that infants who show decreased heart rate from baseline to task also show enhanced performance on the A-not-B task. Studies utilizing the heart rate phases (Richards and Casey, 1992) during infant working memory tasks would provide greater insight into the effects of sustained attention on working memory performance.

Relations between arousal and attention are complex and change throughout development. The significant and sustained decrease in heart rate associated with attention is most likely limited to infancy and early childhood; however, individual differences in heart rate variability are related to attention and cognitive performance throughout development (Porges, 1992; Suess et al., 1994; Reynolds and Richards, 2008). Relatively little work has examined the influence of arousal aspects of attention on working memory in later development. An exception would be the work by Thayer and colleagues (Hansen et al., 2003; Thayer et al., 2009) examining relations between HRV and working memory in adults. Their findings indicate that individual differences in baseline HRV are associated with performance on working memory tasks. Individuals with high baseline HRV perform better on working memory tasks than individuals with low baseline HRV, and the advantage is specific to tasks requiring executive function (Thayer et al., 2009). Thus, attention and arousal appear to influence working memory throughout development; however, the dynamics of these relations are complex and would be expected to change significantly with age.

The development of attention and the development of working memory are closely related. Significant gains on working memory tasks overlap in developmental timing with key periods for development of sustained attention, the posterior orienting system, and the anterior attention system. There is also significant overlap in neural systems involved in attention and working memory. The cortical sources of the Nc ERP component associated with infant visual attention have been localized to areas of PFC (Reynolds and Richards, 2005; Reynolds et al., 2010). Similarly, research with fNIRS indicates that frontal and parietal areas are involved in working memory performance for infants (Baird et al., 2002) and preschoolers (Buss et al., 2014). Given the substantial overlap in developmental timing and neural systems involved in both attention and working memory, future research should aim to examine relations between attention and working memory in infancy and early childhood using both psychophysiological and neural measures. A multi-level analysis approach would be ideal for addressing the controversy regarding the relative contribution of PFC, parietal cortex, and medial temporal lobe structures to working memory performance. Attention plays a key role in successful working memory performance, and the development of attention systems most likely influences the development of working memory. Bidirectional effects are common throughout development, and thus of equal interest is the potential influence of working memory on further development of attention systems in infancy and early childhood.

## Author Contributions

After discussions about potential directions for the article, the authors (GDR and ACR) settled on the overall content to include and outline to follow for the article. ACR provided recommendations on potential content for several of the major sections of the article. GDR incorporated much of ACR’s work into the article when he wrote the original draft, and subsequently incorporated further input from ACR into the final version of the manuscript.

## Conflict of Interest Statement

The authors declare that the research was conducted in the absence of any commercial or financial relationships that could be construed as a potential conflict of interest.

## References

[B1] Amso D. Scerif G. (2015). The attentive brain: insights from developmental cognitive neuroscience. Nat. Rev. Neurosci. 16, 606–619. 10.1038/nrn4025 26383703 PMC4885514

[B2] Astle D. E. Scerif G. (2011). Interactions between attention and visual short-term memory (VSTM): what can be learnt from individual and developmental differences? Neuropsychologia 49, 1435–1445. 10.1016/j.neuropsychologia.2010.12.001 21185321

[B3] Awh E. Jonides J. (2001). Overlapping mechanisms of attention and spatial working memory. Trends Cogn. Sci. 5, 119–126. 10.1016/s1364-6613(00)01593-x 11239812

[B4] Awh E. Vogel E. K. Oh S. H. (2006). Interactions between attention and working memory. Neuroscience 139, 201–208. 10.1016/j.neuroscience.2005.08.023 16324792

[B5] Bachevalier J. Brickson M. Hagger C. (1993). Limbic-dependent recognition memory in monkeys develops early in infancy. Neuroreport 4, 77–80. 10.1097/00001756-199301000-00020 8453042

[B6] Baddeley A. (1996). The fractionation of working memory. Proc. Natl. Acad. Sci. U S A 93, 13468–13472. 10.1073/pnas.93.24.13468 8942958 PMC33632

[B600] Baird A. A. Kagan J. Gaudette T. Walz K. A. Hershlag N. Boas D. A. (2002). Frontal lobe activation during object permanence: data from near-infrared spectroscopy. Neuroimage 16, 1120–1126. 10.1006/nimg.2002.1170 12202098

[B7] Bauer P. J. (2009). “The cognitive neuroscience of the development of memory,” in The Development of Memory in Infancy and Childhood, eds Courage M. Cowan N. (New York, NY: Psychology Press), 115–144.

[B8] Begleiter H. Porjesz B. Wang W. (1993). A neurophysiologic correlate of visual short-term memory in humans. Electroencephalogr. Clin. Neurophysiol. 87, 46–53. 10.1016/0013-4694(93)90173-s 7687953

[B9] Bell M. A. (2001). Brain electrical activity associated with cognitive processing during a looking version of the A-not-B task. Infancy 2, 311–330. 10.1207/s15327078in0203_2 33451207

[B10] Bell M. A. (2002). Power changes in infant EEG frequency bands during a spatial working memory task. Psychophysiology 39, 450–458. 10.1111/1469-8986.3940450 12212637

[B11] Bell M. A. (2012). A psychobiological perspective on working memory performance at 8 months of age. Child Dev. 83, 251–265. 10.1111/j.1467-8624.2011.01684.x 22103396

[B12] Bell M. A. Adams S. E. (1999). Comparable performance on looking and reaching versions of the A-not-B task at 8 months of age. Infant Behav. Dev. 22, 221–235. 10.1016/s0163-6383(99)00010-7

[B14] Bell M. A. Fox N. A. (1994). “Brain development over the first year of life: relations between EEG frequency and coherence and cognitive and affective behaviors,” in Human Behavior and the Developing Brain, eds Dawson G. Fischer K. (New York, NY: Guilford), 314–345.

[B13] Bell M. A. Wolfe C. D. (2007). Changes in brain functioning from infancy to early childhood: evidence from EEG power and coherence during working memory tasks. Dev. Neuropsychol. 31, 21–38. 10.1207/s15326942dn3101_2 17305436

[B15] Brown M. W. Aggleton J. P. (2001). Recognition memory: what are the roles of the perirhinal cortex and hippocampus? Nat. Rev. Neurosci. 2, 51–61. 10.1038/35049064 11253359

[B16] Buss A. T. Fox N. Boas D. A. Spencer J. P. (2014). Probing the early development of visual working memory capacity with functional near-infrared spectroscopy. Neuroimage 85, 314–325. 10.1016/j.neuroimage.2013.05.034 23707803 PMC3859697

[B17] Colombo J. (2001). The development of visual attention in infancy. Annu. Rev. Psychol. 52, 337–367. 10.1146/annurev.psych.52.1.337 11148309

[B170] Colombo J. Mitchell D. W. (1990). “Individual and developmental differences in infant visual attention,” in Individual Differences in Infancy, eds Colombo J. Fagen J. W. (Hillsdale, NJ: Erlbaum), 193–227.

[B18] Courage M. L. Howe M. L. (2004). Advances in early memory development research: insights about the dark side of the moon. Dev. Rev. 24, 6–32. 10.1016/j.dr.2003.09.005

[B19] Courage M. L. Reynolds G. D. Richards J. E. (2006). Infants’ attention to patterned stimuli: developmental change from 3 to 12 months of age. Child Dev. 77, 680–695. 10.1111/j.1467-8624.2006.00897.x 16686795 PMC1463994

[B20] Courchesne E. Ganz L. Norcia A. M. (1981). Event-related brain potentials to human faces in infants. Child Dev. 52, 804–811. 10.2307/1129080 7285651

[B21] Courtney S. M. Ungerleider L. G. Keil K. Haxby J. V. (1997). Transient and sustained activity in a distributed neural system for human working memory. Nature 386, 608–611. 10.1038/386608a0 9121584

[B22] Cowan N. (1995). Attention and Memory: An Integrated Framework. New York, NY: Oxford University Press.

[B23] Cowan N. Morey C. C. (2006). Visual working memory depends on attentional filtering. Trends Cogn. Sci. 10, 139–141. 10.1016/j.tics.2006.02.001 16497538 PMC2635910

[B24] Crone E. A. Wendelken C. Donohue S. van Leijenhorst L. Bunge S. A. (2006). Neurocognitive development of the ability to manipulate information in working memory. Proc. Natl. Acad. Sci. U S A 103, 9315–9320. 10.1073/pnas.0510088103 16738055 PMC1472660

[B25] Cuevas K. Bell M. A. (2010). Developmental progression of looking and reaching performance on the A-not-B task. Dev. Psychol. 46, 1363–1371. 10.1037/a0020185 20822245 PMC3564966

[B26] Cuevas K. Bell M. A. (2011). EEG and ECG from 5 to 10 months of age: developmental changes in baseline activation and cognitive processing during a working memory task. Int. J. Psychophysiol. 80, 119–128. 10.1016/j.ijpsycho.2011.02.009 21338632 PMC3096566

[B27] Curtis C. E. D’Esposito M. (2003). Persistent activity in the prefrontal cortex during working memory. Trends Cogn. Sci. 7, 415–423. 10.1016/s1364-6613(03)00197-9 12963473

[B29] de Haan M. Nelson C. A. (1997). Recognition of the mother’s face by six-month-old infants: a neurobehavioral study. Child Dev. 68, 187–210. 10.1111/j.1467-8624.1997.tb01935.x 9179998

[B28] de Haan M. Nelson C. A. (1999). Brain activity differentiates face and object processing in 6-month-old infants. Dev. Psychol. 35, 1113–1121. 10.1037/0012-1649.35.4.1113 10442879

[B30] Desimone R. (1996). Neural mechanisms for visual memory and their role in attention. Proc. Natl. Acad. Sci. U S A 93, 13494–13499. 10.1073/pnas.93.24.13494 8942962 PMC33636

[B300] D’Esposito M. Postle B. R. Ballard D. Lease J. (1999). Maintenance versus manipulation of information held in working memory: an event-related fMRI study. Brain cogn. 41, 66–86. 10.1006/brcg.1999.1096 10536086

[B31] Diamond A. (1985). Development of the ability to use recall to guide action, as Indicated by infants’ performance on AB. Child Dev. 56, 868–883. 10.2307/1130099 4042750

[B32] Diamond A. (1990). “Rate of maturation of the hippocampus and the developmental progression of children’s performance on the delayed non-matching to sample and visual paired comparison tasks,” in Development and Neural Bases of Higher Cognitive Functions, ed. Diamond A. (New York, NY: New York Academy of Sciences Press), 394–426. 10.1111/j.1749-6632.1990.tb48904.x 2127514

[B33] Eichenbaum H. Yonelinas A. Ranganath C. (2007). The medial temporal lobe and recognition memory. Annu. Rev. Neurosci. 30, 123–152. 10.1146/annurev.neuro.30.051606.094328 17417939 PMC2064941

[B34] Fahy F. L. Riches I. P. Brown M. W. (1993). Neuronal activity related to visual recognition memory: long-term memory and the encoding of recency and familiarity information in the primate anterior and medial inferior rhinal cortex. Exp. Brain Res. 96, 457–472. 10.1007/bf00234113 8299747

[B35] Freeseman L. J. Colombo J. Coldren J. T. (1993). Individual differences in infant visual attention: four-month-olds’ discrimination and generalization of global and local stimulus properties. Child Dev. 64, 1191–1203. 10.2307/1131334 8404264

[B36] Frick J. E. Richards J. E. (2001). Individual differences in infants’ recognition of briefly presented visual stimuli. Infancy 2, 331–352. 10.1207/s15327078in0203_3 33451214

[B37] Fuster J. M. (1997). The Prefrontal Cortex: Anatomy, Physiology, and Neuropsychology of the Frontal Lobes. New York: Raven Press.

[B38] Gilmore R. Johnson M. H. (1995). Working memory in infancy: six-month-olds^′^ performance on two versions of the oculomotor delayed response task. J. Exp. Child Psychol. 59, 397–418. 10.1006/jecp.1995.1019 7622986

[B39] Goldman-Rakic P. S. (1995). Cellular basis of working memory. Neuron 14, 477–485. 10.1016/0896-6273(95)90304-6 7695894

[B40] Guy M. W. Reynolds G. D. Zhang D. (2013). Visual attention to global and local stimulus properties in six-month-old infants: individual differences and event-related potentials. Child Dev. 84, 1392–1406. 10.1111/cdev.12053 23379931

[B41] Guy M. W. Zieber N. Richards J. E. (in press). The cortical development of specialized face processing in infancy. Child Dev. 84, 1392–1406. 10.1111/cdev.12053 27246260 PMC5042801

[B42] Hansen A. L. Johnsen B. H. Thayer J. F. (2003). Vagal influence on working memory and attention. Int. J. Psychophysiol. 48, 263–274. 10.1016/s0167-8760(03)00073-4 12798986

[B43] Hofstadter M. Reznick J. S. (1996). Response modality affects human infant delayed-response performance. Child Dev. 67, 646–658. 10.2307/1131838 8625732

[B44] Hood B. M. (1995). Shifts of visual attention in the human infant: a neuroscientific approach. Adv. Infancy Res. 10, 163–216.

[B45] Hunter M. Ames E. (1988). “A multifactor model of infant preferences for novel and familiar stimuli,” in Advances in Infancy Research, (Vol. 5), eds Rovee-Collier C. Lipsitt L. P. (Norwood, NJ: Ablex), 69–95.

[B47] Johnson M. H. Posner M. Rothbart M. K. (1991). Components of visual orienting in early infancy: contingency learning, anticipatory looking and disengaging. J. Cogn. Neurosci. 3, 335–344. 10.1162/jocn.1991.3.4.335 23967813

[B52] Káldy Z. Guillory S. Blaser E. (2015). Delayed match retrieval: a novel anticipation-based visual working memory paradigm. Dev. Sci. [Epub ahead of print]. 10.1111/desc.12335 26234951 PMC4733613

[B49] Káldy Z. Leslie A. M. (2003). Identification of objects in 9-month-old infants: integrating ‘what’and ‘where’information. Dev. Sci. 6, 360–373. 10.1111/1467-7687.00290

[B50] Káldy Z. Leslie A. M. (2005). A memory span of one? Object identification in 6.5-month-old infants. Cognition 97, 153–177. 10.1016/j.cognition.2004.09.009 16226561

[B51] Káldy Z. Sigala N. (2004). The neural mechanisms of object working memory: what is where in the infant brain? Neurosci. Biobehav. Rev. 28, 113–121. 10.1016/j.neubiorev.2004.01.002 15172760

[B53] Kane M. J. Engle R. W. (2002). The role of prefrontal cortex in working-memory capacity, executive attention and general fluid intelligence: an individual differences perspective. Psychon. Bull. Rev. 9, 637–671. 10.3758/bf03196323 12613671

[B54] Klingberg T. Forssberg H. Westerberg H. (2002). Increased brain activity in frontal and parietal cortex underlies the development of visuospatial working memory capacity in childhood. J. Cogn. Neurosci. 14, 1–10. 10.1162/089892902317205276 11798382

[B56] Li L. Miller E. K. Desimone R. (1993). The representation of stimulus familiarity in anterior inferior temporal cortex. J. Neurophysiol. 69, 1918–1929. 8350131 10.1152/jn.1993.69.6.1918

[B57] Luciana M. Nelson C. A. (1998). The functional emergence of prefrontally-guided memory systems in four- to eight-year-old children. Neuropsychologia 36, 272–293. 10.1016/s0028-3932(97)00109-7 9622192

[B60] Nelson C. A. (1995). The ontogeny of human memory: a cognitive neuroscience perspective. Developmental Psychology 5, 723–738. 10.1002/9780470753507.ch10

[B64] Pelphrey K. A. Reznick J. S. (2003). “Working memory in infancy,” in Advances in Child Behavior, (Vol. 31), ed. Kail R. V. (San Diego, CA: Academic Press), 173–227. 10.1016/s0065-2407(03)31005-5 14528662

[B63] Pelphrey K. A. Reznick J. S. Davis Goldman B. Sasson N. Morrow J. Donahoe A.. (2004). Development of visuospatial short-term memory in the second half of the 1st year. Dev. Psychol. 40, 836–851. 10.1037/0012-1649.40.5.836 15355170

[B67] Perone S. Simmering V. Spencer J. (2011). Stronger neural dynamics capture changes in infants’ visual working memory capacity over development. Dev. Sci. 14, 1379–1392. 10.1111/j.1467-7687.2011.01083.x 22010897 PMC3665414

[B66] Perone S. Spencer J. P. (2013a). Autonomy in action: linking the act of looking to memory formation in infancy via dynamic neural fields. Cogn. Sci. 37, 1–60. 10.1111/cogs.12010 23136815 PMC3815444

[B65] Perone S. Spencer J. P. (2013b). Autonomous visual exploration creates developmental change in familiarity and novelty seeking behaviors. Front. Psychol. 4:648. 10.3389/fpsyg.2013.00648 24065948 PMC3778377

[B690] Porges S. W. (1992). “Autonomic regulation and attention,” in Attention and Information Processing in Infants and Adults: Perspectives from Human and Animal Research, eds Campbell B. A. Hayne H. Richardson R. (Hillsdale, NJ: Lawrence Erlbaum Associates), 201–223.

[B69] Posner M. I. Peterson S. (1990). The attention system of the human brain. Annu. Rev. Neurosci. 13, 25–42. 10.1146/annurev.neuro.13.1.25 2183676

[B73] Reynolds G. D. (2015). Infant visual attention and object recognition. Behav. Brain Res. 285, 34–43. 10.1016/j.bbr.2015.01.015 25596333 PMC4380660

[B71] Reynolds G. D. Courage M. L. Richards J. E. (2010). Infant attention and visual preferences: converging evidence from behavior, event-related potentials and cortical source localization. Dev. Psychol. 46, 886–904. 10.1037/a0019670 20604609 PMC3961705

[B72] Reynolds G. D. Courage M. L. Richards J. E. (2013). “The development of attention,” in Oxford Handbook of Cognitive Psychology, ed. Reisberg D. (New York, NY: Oxford University Press), 1000–1013.

[B720] Reynolds G. D. Guy M. W. Zhang D. (2011). Neural correlates of individual differences in infant visual attention and recognition memory. Infancy 16, 368–391. 10.1111/j.1532-7078.2010.00060.x 21666833 PMC3110012

[B70] Reynolds G. D. Richards J. E. (2005). Familiarization, attention and recognition memory in infancy: an ERP and cortical source localization study. Dev. Psychol. 41, 598–615. 10.1037/0012-1649.41.4.598 16060807 PMC1464099

[B74] Reynolds G. D. Richards J. E. (2008). “Infant heart rate: a developmental psychophysiological perspective,” in Developmental Psychophysiology: Theory, Systems and Applications, eds Schmidt L. A. Segalowitz S. J. (Cambridge: Cambridge University Press), 173–212.

[B75] Reznick J. S. Morrow J. D. Goldman B. D. Snyder J. (2004). The onset of working memory in infants. Infancy 6, 145–154. 10.1207/s15327078in0601_7

[B750] Richards J. E. (1985). The development of sustained visual attention in infants from 14 to 26 weeks of age. Psychophysiology 22, 409–416. 10.1111/j.1469-8986.1985.tb01625.x 4023152

[B76] Richards J. E. (1997). Effects of attention on infants’ preference for briefly exposed visual stimuli in the paired-comparison recognition-memory paradigm. Dev. Psychol. 33, 22–31. 10.1037/0012-1649.33.1.22 9050387

[B77] Richards J. E. (2003). Attention affects the recognition of briefly presented visual stimuli in infants: an ERP study. Dev. Sci. 6, 312–328. 10.1111/1467-7687.00287 16718304 PMC1464402

[B80] Richards J. E. (2008). “Attention in young infants: a developmental psychophysiological perspective,” in Handbook of Developmental Cognitive Neuroscience, eds Nelson C. A. Luciana M. (Cambridge, MA: MIT Press), 479–497.

[B81] Richards J. E. (2010). “Attention in the brain and early infancy,” in Neoconstructivism: The New Science of Cognitive Development, ed. Johnson S. P. (New York, NY: Oxford University Press), 3–31.

[B82] Richards J. E. Casey B. J. (1992). “Development of sustained visual attention in the human infant,” in Attention and Information Processing in Infants and Adults: Perspectives from Human and Animal Research, eds Campbell B. A. Hayne H. (Hillsdale, NJ: Erlbaum Publishing), 30–60.

[B78] Richards J. E. Cronise K. (2000). Extended visual fixation in the early preschool years: look duration, heart rate changes and attentional inertia. Child Dev. 71, 602–620. 10.1111/1467-8624.00170 10953928

[B79] Richards J. E. Turner E. D. (2001). Extended visual fixation and distractibility in children from six to twenty-four months of age. Child Dev. 72, 963–972. 10.1111/1467-8624.00328 11480948

[B83] Rose S. A. Feldman J. F. Jankowski J. J. (2004). Infant visual recognition memory. Dev. Rev. 24, 74–100. 10.1016/j.dr.2003.09.004 12760523

[B84] Rose S. A. Gottfried A. W. Melloy-Carminar P. M. Bridger W. H. (1982). Familiarity and novelty preferences in infant recognition memory: implications for information processing. Dev. Psychol. 18, 704–713. 10.1037/0012-1649.18.5.704

[B86] Ross-Sheehy S. Oakes L. M. Luck S. J. (2003). The development of visual short-term memory capacity in infants. Child Dev. 74, 1807–1822. 10.1046/j.1467-8624.2003.00639.x 14669897

[B85] Ross-Sheehy S. Oakes L. M. Luck S. J. (2011). Exogenous attention influences visual short-term memory in infants. Dev. Sci. 14, 490–501. 10.1111/j.1467-7687.2010.00992.x 21477189 PMC3076103

[B87] Rovee-Collier C. Cuevas K. (2009). Multiple memory systems are unnecessary to account for infant memory development: an ecological model. Dev. Psychol. 45, 160-174. 10.1037/a0014538 19209999 PMC2693033

[B88] Ruff H. A. Capozzoli M. C. (2003). Development of attention and distractibility in the first 4 years of life. Dev. Psychol. 39, 877–890. 10.1037/0012-1649.39.5.877 12952400

[B89] Ruff H. A. Rothbart M. K. (1996). Attention in Early Development. New York: Oxford University Press.

[B90] Sarter M. Givens B. Bruno J. P. (2001). The cognitive neuroscience of sustained attention: where top-down meets bottom-up. Brain Res. Brain Res. Rev. 35, 146–160. 10.1016/s0165-0173(01)00044-3 11336780

[B91] Scherf K. S. Sweeney J. A. Luna B. (2006). Brain basis of developmental change in visuospatial working memory. J. Cogn. Neurosci. 18, 1045–1058. 10.1162/jocn.2006.18.7.1045 16839280

[B92] Schiller P. H. (1985). “A model for the generation of visually guided saccadic eye movements,” in Models of the Visual Cortex, eds Rose D. Dobson V. G. (New York, NY: Wiley), 62–70.

[B930] Simmering V. R. (2012). The development of visual working memory capacity during early childhood. J. Exp. Child. Psychol. 111, 695–707. 10.1016/j.jecp.2011.10.007 22099167

[B920] Simmering V. R. Schutte A. R. Spencer J. P. (2008). Generalizing the dynamic field theory of spatial cognition across real and developmental time scales. Brain Res. 1202, 68–86. 10.1016/j.brainres.2007.06.081 17716632 PMC2593104

[B910] Simmering V. R. Spencer J. P. (2008). Generality with specificity: the dynamic field theory generalizes across tasks and time scales. Dev. Sci. 11, 541–555. 10.1111/j.1467-7687.2008.00700.x 18576962 PMC2593101

[B93] Smith L. B. Thelen E. Titzer R. McLin D. (1999). Knowing in the context of acting: the task dynamics of the A-not-B error. Psychol. Rev. 106, 235-260. 10.1037/0033-295x.106.2.235 10378013

[B94] Snyder K. (2010). Neural correlates of encoding predict infants’ memory in the paired-comparison procedure. Infancy 15, 270–299. 10.1111/j.1532-7078.2009.00015.x 32693544

[B95] Sokolov E. N. (1963). Perception and the Conditioned Reflex. Oxford: Pergamon Press.

[B960] Spencer J. P. Simmering V. R. Schutte A. R. Schöner G. (2007). “What does theoretical neuroscience have to offer the study of behavioral development? Insights from a dynamic field theory of spatial cognition,” in The Emerging Spatial Mind, eds Plumert J. Spencer J. P. (New York, NY: Oxford University Press), 320–321.

[B96] Stedron J. M. Sahni S. D. Munakata Y. (2005). Common mechanisms for working memory and attention: the case of perseveration with visible solutions. J. Cogn. Neurosci. 17, 623–631. 10.1162/0898929053467622 15829082

[B9600] Suess P. E. Porges S. W. Plude D. J. (1994). Cardiac vagal tone and sustained attention in school-age children. Psychophysiology 31, 17–22. 10.1111/j.1469-8986.1994.tb01020.x 8146250

[B97] Sweeney J. A. Mintun M. A. Kwee S. Wiseman M. B. Brown D. L. Rosenberg D. R.. (1996). Positron emission tomography study of voluntary saccadic eye movements and spatial working memory. J. Neurophysiol. 75, 454–468. 8822570 10.1152/jn.1996.75.1.454

[B98] Thayer J. F. Hansen A. L. Saus-Rose E. Johnsen B. H. (2009). Heart rate variability, prefrontal neural function and cognitive performance: the neurovisceral integration perspective on self-regulation, adaptation and health. Ann. Behav. Med. 37, 141–153. 10.1007/s12160-009-9101-z 19424767

[B99] Wan H. Aggleton J. P. Brown M. W. (1999). Different contributions of the hippocampus and perirhinal cortex to recognition memory. J. Neurosci. 19, 1142–1148. 9920675 10.1523/JNEUROSCI.19-03-01142.1999 PMC6782155

[B990] Wiggs C. L. Martin A. (1998). Properties and mechanisms of perceptual priming. Curr. Opin. Neurobiol. 8, 227–233. 10.1016/S0959-4388(98)80144-X 9635206

[B100] Xiang J.-Z. Brown M. W. (1998). Differential neuronal encoding of novelty, familiarity and recency in regions of the anterior temporal lobe. Neuropharmacology 37, 657–676. 10.1016/s0028-3908(98)00030-6 9705004

[B101] Zeamer A. Heuer E. Bachevalier J. (2010). Developmental trajectory of object recognition in infant rhesus macaques with and without neonatal hippocampal lesions. J. Neurosci. 30, 9157–9165. 10.1523/JNEUROSCI.0022-10.2010 20610749 PMC2913301

[B102] Zhu X. O. Brown M. W. McCabe B. J. Aggleton J. P. (1995). Effects of the novelty or familiarity of visual stimuli on the expression of the intermediate early gene c-fos in the rat brain. Neuroscience 69, 821–829. 10.1016/0306-4522(95)00320-i 8596651

